# Protein phosphatase 2A (PP2A) inhibitor CIP2A indicates resistance to radiotherapy in rectal cancer

**DOI:** 10.1002/cam4.1361

**Published:** 2018-02-14

**Authors:** Eva‐Maria Birkman, Adam Elzagheid, Terhi Jokilehto, Tuulia Avoranta, Eija Korkeila, Jarmo Kulmala, Kari Syrjänen, Jukka Westermarck, Jari Sundström

**Affiliations:** ^1^ Department of Pathology University of Turku Turku Finland; ^2^ Department of Pathology Turku University Hospital Turku Finland; ^3^ Department of Pathology Faculty of Medicine Benghazi University Benghazi Libya; ^4^ Department of Genetic Engineering Biotechnology Research Center Tripoli Libya; ^5^ Department of Medical Biochemistry and Genetics University of Turku Turku Finland; ^6^ Department of Oncology University of Turku and Turku University Hospital Turku Finland; ^7^ Department of Clinical Research Biohit Oyj Helsinki Finland; ^8^ Molecular Oncology Research Center Barretos Cancer Hospital Barretos Brazil; ^9^ Turku Centre for Biotechnology University of Turku and Åbo Akademi University Turku Finland

**Keywords:** Cancerous inhibitor of protein phosphatase 2A, chemoradiotherapy, rectal cancer

## Abstract

Preoperative (chemo)radiotherapy, (C)RT, is an essential part of the treatment of rectal cancer patients, but tumor response to this therapy among patients is variable. Thus far, there are no clinical biomarkers that could be used to predict response to (C)RT or to stratify patients into different preoperative treatment groups according to their prognosis. Overexpression of cancerous inhibitor of protein phosphatase 2A (CIP2A) has been demonstrated in several cancers and is frequently associated with reduced survival. Recently, high CIP2A expression has also been indicated to contribute to radioresistance in head and neck squamous cell carcinoma, but few studies have examined the connection between CIP2A and radiation response regarding other malignancies. We have evaluated CIP2A protein expression levels in relation to tumor regression after preoperative (C)RT and survival of rectal adenocarcinoma patients. The effects of *CIP2A* knockdown by siRNA on cell survival were further investigated in colorectal cancer cells exposed to radiation. Patients with low‐CIP2A‐expressing tumors had more frequently moderate or excellent response to long‐course (C)RT than patients with high‐CIP2A‐expressing tumors. They also had higher 36‐month disease‐specific survival (DSS) rate in categorical analysis. In the multivariate analysis, low CIP2A expression level remained as an independent predictive factor for increased DSS. Suppression of *CIP2A* transcription by siRNA was found to sensitize colorectal cancer cells to irradiation and decrease their survival in vitro. In conclusion, these results suggest that by contributing to radiosensitivity of cancer cells, low CIP2A protein expression level associates with a favorable response to long‐course (C)RT in rectal cancer patients.

## Introduction

Colorectal cancer (CRC) is the third most common cancer worldwide 1, 2, and approximately one‐third of the incidence is accounted for rectal cancer. Preoperative radiotherapy (RT) is used for a part of rectal cancer patients to reduce the risk of local recurrence or to enable surgery for initially nonresectable tumors. It can be given either as short‐course RT or long‐course chemoradiotherapy (CRT) with 5‐fluorouracil (5‐FU) depending on clinical staging. The latter is usually recommended for locally advanced rectal cancer 3.

However, the response to preoperative therapy in terms of tumor regression or disease outcome is variable, and some patients might benefit from a more individualized risk‐benefit assessment than is currently available 4, 5, 6. Thus far, there are no clinical molecular markers that can aid in predicting response to (C)RT or enable stratifying patients into separate treatment groups according to their prognosis 3. Therefore, various factors have been investigated in order to determine their predictive or prognostic potential regarding (C)RT. These factors include for example markers associated with cell proliferation, apoptosis, angiogenesis, inflammatory response, diverse oncogenic pathways, epithelial‐mesenchymal transition, microsatellite instability, or the stem‐cell nature of cancer cells 6, 7, 8, 9, 10, 11, 12, 13, 14, 15, 16.

CIP2A (cancerous inhibitor of PP2A) inhibits protein phosphatase 2A (PP2A) tumor suppressor activity and thereby promotes malignant cell transformation and tumor growth 17, 18, 19. CIP2A is known to be overexpressed in several cancer types, and its overexpression has been demonstrated to associate with reduced survival for example in gastric cancer 20, serous ovarian carcinoma 21, nonsmall‐cell lung carcinoma 22, chronic myeloid leukemia 23, CRC 24, and head and neck squamous cell carcinoma (HNSCC) 25. CIP2A expression has also been linked to poorly differentiated histology in various cancers 18, 19, 26, 27. CIP2A promotes several signaling pathways contributing to cell proliferation, apoptosis resistance, and senescence evasion. In addition to Myc, CIP2A‐mediated inhibition of protein phosphatase 2A (PP2A) is known to stimulate Akt kinase and E2F transcription factor 1 (E2F1) pathways 18, 19. In normal tissues, except testis, CIP2A is expressed only at a low level, and its inhibition does not impair normal development or viability in mice 19, 28.

In connection to irradiation, CIP2A expression has been previously studied in radiated mouse testes and found to be expressed in radioresistant testicular stem‐cell population, where it is targeted by octamer‐binding transcription factor 4 (Oct4). The cooperation of CIP2A and Oct4 was shown to contribute to radioresistance in HNSCC cell lines and tumorigenicity in HNSCC xenograft models 25. Importantly, the in vivo relevance of CIP2A in mediating intestinal radioresistance was recently demonstrated when CIP2A was shown to physically interact with Myc and support Myc‐mediated intestinal regeneration in response to irradiation in vivo 29.

In this study, we have examined the relationship between CIP2A expression and radiation response both by immunohistochemistry (IHC) in clinical samples collected from rectal cancer patients and in vitro by assessing the effects of *CIP2A* knockdown on cell survival in a CRC cell line exposed to radiation.

## Material and Methods

### Patients and tumor samples

The study population consisted of 210 rectal cancer patients with tumors located in either middle or distal rectum. They were operated at Turku University Hospital between 2000 and 2009. Representative formalin‐fixed paraffin‐embedded (FFPE) surgical specimens were retrieved from the archives of the Department of Pathology, Turku University Hospital, and full clinicopathologic data were retrospectively gathered from those patients. Superficial tumors operated by local excision were excluded from the study as well as tumors from patients with distant metastases at the time of diagnosis. Pretreatment biopsies from patients treated with preoperative radiotherapy were also collected but not included in the analyses due to the small number of samples (*n *=* *10).

The patients received either short‐course preoperative RT (*n *=* *89, 42%), long‐course preoperative (C)RT (*n *=* *51, 24%), or no treatment before surgery (*n *=* *70, 33%), according to common clinical recommendations 30. The type of treatment was chosen based on preoperative tumor staging, which included computed tomography (CT) or magnetic resonance imaging (MRI) of the rectum, abdominal CT, and chest X‐ray or CT.

Short‐course RT consisted of a total dose of 25 Gy delivered over 5 days in 5 Gy fractions, and the patients were operated on the following week. Long‐course RT was given in 1.8 Gy fractions to a total dose of 50.4 Gy over 6 weeks with (*n *=* *43) or without (*n *=* *8) chemotherapy, and the patients were operated five to 7 weeks after RT. Chemotherapy included either bolus 5‐fluorouracil (5‐FU, *n *=* *5) or capecitabine (*n *=* *38). The type of surgery was anterior resection among 113 (54%) patients and abdominoperineal resection among 94 patients (45%). Three patients (1%) were operated with low Hartmann's procedure or other type of surgery. Lymphovascular invasion was found in 45 of 157 (29%) tumors. Patients with established high‐risk features were treated with adjuvant chemotherapy.

Tumors were staged according to the TNM criteria applicable at the time of surgery 31. Follow‐up information was available for 206 patients. The median follow‐up time was 74.5 months, and disease recurrence (local or distant) was observed among 67 patients (33%). The basic clinicopathologic characteristics are shown in Table 1.

**Table 1:** Patient characteristics (*n *=* *210)

	Short‐course RT, *n* (%)	Long‐course RT, *n* (%)	Control, *n* (%)
Sex			
Male	55 (45.5)	33 (27.3)	33 (27.3)
Female	34 (38.2)	18 (20.2)	37 (41.6)
Mean age (years)	65.3	64.3	73.9
Median age (years)	68.0	66.1	74.1
Range	34.3–82.0	41.9–81.2	47.0–92.3
Preoperative T^a^			
T1‐2	27 (30.0)	0 (0)	21 (30.0)
T3	54 (60.7)	2 (3.9)	12 (17.1)
T4a	1 (1.1)	46 (90.2)	3 (4.3)
Tx	7 (7.9)	1 (2.0)	34 (48.6)
Postoperative T			
T1	3 (3.4)	2 (3.9)	5 (7.1)
T2	32 (36.0)	7 (13.7)	26 (37.1)
T3	50 (56.2)	28 (54.9)	36 (51.4)
T4^b^	3 (3.4)	13 (25.5)	3 (4.3)
T0	1 (1.1)	1 (2.0)	0 (0)
Postoperative N			
N0	52 (58.4)	32 (62.7)	39 (55.7)
N1	25 (28.1)	14 (27.5)	16 (22.9)
N2	12 (13.5)	5 (9.8)	12 (17.1)
Nx	0 (0)	0 (0)	3 (4.3)
Postoperative stage			
Stage I	26 (29.2)	5 (9.8)	25 (35.7)
Stage II	26 (29.2)	27 (52.9)	17 (24.3)
Stage III	36 (40.4)	18 (35.3)	28 (40.0)
No vital tumor	1 (1.1)	1 (2.0)	0 (0)
Postoperative grade			
G1	9 (10.1)	10 (19.6)	13 (18.6)
G2	56 (62.9)	33 (64.7)	46 (65.7)
G3	22 (24.7)	3 (5.9)	11 (15.7)
Gx	2 (2.2)	5 (9.8)	0 (0)
Circumferential margin (mm)			
0	3 (3.4)	8 (15.7)	4 (5.7)
0 ≤ CRM ≤ 2	8 (9.0)	9 (17.6)	7 (10.0)
>2	65 (73.0)	22 (43.1)	30 (42.9)
Unknown	13 (14.6)	12 (23.5)	29 (41.4)
Follow‐up status			
Alive without disease	56 (62.9)	19 (37.3)	30 (42.9)
Alive with disease	2 (2.2)	3 (5.9)	4 (5.7)
Died of disease	19 (21.3)	20 (39.2)	20 (28.6)
Died of other causes	12 (13.5)	9 (17.6)	16 (22.9)

RT, radiotherapy; CRM, circumferential resection margin.

a Missing information for two patients in the long‐course RT group.

b Includes T3 tumors with threatened circumferential margin involvement.

The study was conducted in accordance with the Declaration of Helsinki and the Finnish legislation for the use of archived tissue specimens and associated clinical information. The clinical data were retrieved, and the histological samples were collected and analyzed with the endorsement of the National Supervisory Authority for Welfare and Health, Finland (Dnro 1709/32/300/02, May 13, 2002).

### Evaluation of the tumor regression grade

Tumor regression after long‐course (C)RT was determined by JS and EK according to a simplified classification based on Dworak and Rödel scales 32, 33, 34, 35. The response to RT was divided into three categories: poor (only minimal or no tumor regression), moderate (some detectable vital tumor cells or cell groups), or excellent response (very few or no detectable tumor cells).

### Immunohistochemistry

The most optimal FFPE tissue samples were selected to obtain enough tumor material for analyses. Antigen retrieval was performed by heating the 5‐μm sections in microwave oven in Target Retrieval buffer, pH 9.0 (Dako Denmark A/S, Glostrup, Denmark) twice for 7 min. The rabbit polyclonal CIP2A antibody 36 was diluted to 1:4000 in Antibody Diluent Buffer (Dako Denmark A/S, Glostrup, Denmark) and incubated for 1 h at RT. For detection, Dual Link System—HRP and DAB Chromogen System (Dako Denmark A/S, Glostrup, Denmark) were used. The stainings were performed with Lab Vision Autostainer.

### Analysis of CIP2A expression

**Figure 1:**
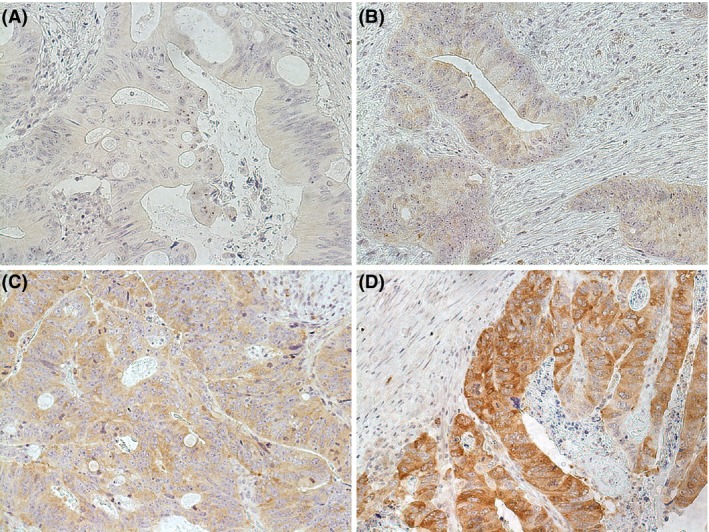
Different CIP2A immunohistochemical cytoplasmic staining intensities in rectal adenocarcinomas. (A) Negative for CIP2A protein expression. (B) Weak (1 + ) CIP2A protein expression. (C) Moderate (2 + ) CIP2A protein expression. (D) Strong (3 + ) CIP2A protein expression. Original objective magnification 20x.

Tumor samples from 204 patients were included in the final analyses. Six tumors could not be reliably evaluated with IHC due to the limited amount of tumor cells. Two observers evaluated the cytoplasmic IHC staining of CIP2A blinded to clinical data (AE all samples and EB 40 samples). Tissue from normal testis was used as a positive control. Cytoplasmic staining was scored as negative (0), weak (1 + ), moderate (2 + ), or strong (3 + ). Strong staining intensity corresponded to the positive control, weak staining intensity was still distinguishable from the background, and moderate staining intensity was intermediate between these two. Representative immunostainings of CIP2A are shown in Figure 1.

For statistical analyses, the most intense cytoplasmic staining index (MICI) and average cytoplasmic staining index (ACI) were used to classify the samples into two subgroups according to the index value being either below or above median level 37. The indices were calculated with the following formula: I = 0 × f0 + 1 × f1 + 2 × f2 + 3 × f3, where I is the staining index and f0–f3 the fractions of cells (from 0 to 1) showing a defined level of staining (from 0 to 3). To obtain MICI, the area containing the most intense staining of cancer cells was chosen from each sample, and the fraction of cancer cells (percentage/100) belonging to each staining intensity category was estimated from that area. ACI was calculated as an average of three randomly selected areas from which the fraction of cancer cells belonging to each staining intensity category was estimated. Theoretically, the indices could vary between 0 and 3. The samples were observed with 10× objective magnification.

### Cell culture experiments and irradiation studies

The RKO CRC cell line (ATCC^®^ CRL‐2577^™^) was purchased from ATCC (Manassas, VA). Cells were grown in Dulbecco's minimal essential medium supplemented with 10% FBS, 2 mmol/L glutamine, and 1% penicillin/streptomycin.

Cells in the logarithmic growth phase were transfected with CIP2A or scrambled double‐stranded small interfering RNAs (siRNA) using Oligofectamine (Invitrogen^™^, Thermo Fisher Scientific, Waltham, MA) according to the manufacturer's instructions. The siRNA sequences have been previously published 26. The cells were lysed in RIPA buffer, and CIP2A protein levels were analyzed by Western blotting with CIP2A antibody (2G10‐3B5; Santa Cruz Biotechnology, Dallas, TX) as described previously 38. HRP‐conjugated anti‐GAPDH mAb (mAbcam 9484; Abcam, Cambridge, UK) was used as a loading control.

Radiation experiments were performed using the 96‐well clonogenic cell survival assay as previously described 39. Forty‐eight hours after the siRNA transfections, the cells were harvested into single‐cell suspensions. The cells were irradiated at room temperature in separate Falcon tubes containing 25 000 cells/treatment in 6 mL culture medium (4167 cells/mL). The irradiation was performed at the radiotherapy department using a linear accelerator (Clinac 2100; Varian CA) with 6 MeV photon irradiation at a dose rate of 2 Gy/min.

After irradiation, the cells were further diluted into 50 mL culture medium in appropriate concentration and 200 μL of cell suspension/well was pipetted in duplicate into 96‐well plates. The number of cells per well was adjusted according to the expected cell death as follows: control 2 cells/well, 0.75 Gy 3 cells/well, 1.25 Gy 4 cells/well, 2.5 Gy 8 cells/well, 5 Gy 20 cells/well, and 7.5 Gy 100 cells/well. The plates were incubated in the cell culture incubator until visible colonies were formed. The plates were examined using an inverted phase contrast microscope. Wells containing colonies of at least 32 cells were considered positive.

The surviving fractions (SF) were calculated with the formula:

$$\text{SF}\;=\;\frac{\text{no. of positive wells/no. of plated cells in control}}{\text{no. of positive wells in control}}$$

The survival curves of cancer cells were fitted using the linear quadratic (LQ) model (SF = exp[‐(αD + βD^2^)]); D, radiation dose). The area under the curve (AUC) values were calculated with a numerical integration algorithm. The results were calculated from three experiments for each treatment with duplicate plates for each radiation dose.

### Statistical analysis

Statistical analyses were performed with IBM SPSS Statistics for Windows, version 21.0 (IBM Corporation, Armonk, NY). Frequency table data were calculated with the Pearson's chi‐squared and Fisher's exact tests. 2 × 2 tables were used to calculate odds ratios (OR) and 95% confidence intervals (CI) using the exact method. Interobserver reproducibility of the IHC assessments was tested with weighted kappa, which was calculated with the intraclass correlation coefficient (ICC) test in parallel mode with a two‐way random model using consistency assumption and the average measures option. The interobserver reproducibility was very good for MICI (weighted kappa 0.83, 95% CI: 0.67–0.91) and moderate for ACI (weighted kappa 0.56, 95% CI: 0.16–0.77).

Univariate survival analysis for disease‐free survival (DFS) and disease‐specific survival (DSS) were performed using the Kaplan–Meier method and log‐rank test. DFS was calculated from the date of diagnosis to disease recurrence or death from any cause, and the observations were censored at the date of last follow‐up. DSS was measured from the date of diagnosis to the date of death from rectal cancer, and the observations were censored at death from other causes or the date of last follow‐up. Cox's proportional hazards regression model was used for multivariate survival analysis, and covariates were entered in a stepwise backward manner. For the irradiation experiments, calculations were performed with Microsoft Excel 2007 (Microsoft Corporation, Redmond, WA) and paired *t*‐test was used to compare the mean AUC values. All statistical tests were two‐sided, and *P*‐values under 0.05 were considered statistically significant.

## Results

### CIP2A expression in relation to clinicopathologic variables

CIP2A MICI was found to associate with patient age at the time of diagnosis and with the depth of tumor invasion (pT). Low CIP2A MICI (≤median, cut‐off 1.20) was more frequently observed among the younger patients (age ≤ median, cut‐off 70 years; Fisher's exact test, *P *=* *0.023; OR 1.98, 95% CI: 1.13–3.47) and among patients with most invasive tumors (pT4 vs. pT1–pT2; Fisher's exact test, *P *=* *0.022). No association was found between CIP2A MICI and patient sex, postoperative histological differentiation grade, lymph node status, postoperative stage, circumferential resection margin (CRM, 1 or 2 mm cut‐off), or lymphovascular invasion (yes vs. no). Low CIP2A ACI (≤median, cut‐off 0.94) was observed slightly more often among the well‐differentiated tumors (Fisher's exact test, *P *=* *0.050). The relationship of CIP2A MICI with selected clinicopathologic variables is presented in Table 2 and for CIP2A ACI in Table S1.

**Table 2:** Association of clinicopathologic variables of rectal cancer patients with CIP2A protein expression defined by the most intense cytoplasmic staining index (*n *=* *204)

	CIP2A most intense cytoplasmic staining index		*P*‐value^a^
	Below median, *n* (%)	Above median, *n* (%)	
Age			
≤70 years	67 (57.8)	36 (40.9)	0.023
>70 years	49 (42.2)	52 (59.1)	
Postoperative T^b^			
pT1‐2	36 (31.3)	37 (42.0)	0.022
pT3	63 (54.8)	48 (54.5)	
pT4	16 (13.9)	3 (3.4)	
Post‐treatment tumor regression^c^			
Poor	15 (42.9)	11 (91.7)	0.006
Moderate/ excellent	20 (57.1)	1 (8.3)	
DSS^d^			
≥36 months	95 (90.5)	60 (76.9)	0.014
<36 months	10 (9.5)	18 (23.1)	

a Pearson's chi‐squared or Fisher's exact test.

b Excluding one tumor that could not be assessed for postoperative T.

c Assessed only after long‐course (chemo)radiotherapy.

d Disease‐specific survival, alive versus death of disease.

### CIP2A expression in relation to tumor regression grade

CIP2A MICI in relation to tumor regression was evaluated in 47 samples from patients having received long‐course (C)RT. Among patients who had moderate or excellent response to (C)RT, CIP2A MICI was more frequently below median than in the poorly responding group (Fisher's exact test, *P *=* *0.006; OR 14.7, 95% CI: 1.70–126.4). Similarly, low CIP2A ACI was associated with moderate or excellent treatment response (Fisher's exact test, *P *=* *0.007; RR 1.63, 95% CI: 1.20–2.20). Tumor regression in relation to CIP2A MICI is presented in Table 2 and in relation to CIP2A ACI in Table S1.

### CIP2A expression in relation to survival

Patients with tumors having CIP2A MICI below median had higher 36‐month disease‐specific survival rate than patients with CIP2A above median when DSS was analyzed as a dichotomous variable (Fisher's exact test, *P *=* *0.014; OR 2.85, 95% CI: 1.23–6.59). (Table 2). A similar trend was also observed between low CIP2A expression and higher 36‐month DSS rate in the case of CIP2A ACI (Fisher's exact test, *P *=* *0.060, Table S1). However, when using 60 months as a cut‐off point, the survival difference between CIP2A MICI levels was no longer significant. No significant relationship was found between CIP2A MICI and DFS as a dichotomous variable using either 36 or 60 months as a cut‐off point.

In the univariate survival analysis, CIP2A MICI was not associated with DFS or DSS. The multivariate analysis for DSS was performed with the following covariates: CIP2A MICI (below vs. above median), RT treatment group (short RT or long (C)RT vs no treatment), sex (female vs. male), age (below vs. above median, 70 years as cut‐off), postoperative nodal status (N1 or N2 vs. N0), the presence of lymphovascular invasion (yes vs. no), CRM (≤2 mm vs. >2 mm), and disease recurrence status (yes vs. no). The remaining factors predicting reduced DSS were CIP2A MICI above median (*P *=* *0.014, HR 3.02, 95% CI: 1.26–7.26), age above 70 years (*P *=* *0.002, HR 3.98, 95% CI: 1.65–9.59), long‐course (C)RT (*P *=* *0.040, HR 3.19, 95% CI 1.05–9.66), and disease recurrence (*P *<* *0.0001, HR 291.8, 95% CI: 36.9–2307.1).

The multivariate analysis for DFS was performed with the same variables as DSS excluding disease recurrence status. CIP2A MICI did not remain as an independent predictive factor, whereas age above 70 years (*P *=* *0.026, HR 1.80, 95% CI: 1.07–3.01) and postoperative nodal status N2 were predictive for reduced DFS (*P *=* *0.002, HR 2.88, 95% CI: 1.48–5.62). Results from multivariate analyses are shown in Table 3.

**Table 3:** Cox's multivariate analysis for disease‐free and disease‐specific survival (*n *=* *138)^a^

	Number of patients	Disease‐free survival			Disease‐specific survival		
		*P*‐value^b^	HR	95% CI	*P*‐value^b^	HR	95% CI
Age							
≤70 years (ref)	63	**0.026**	1.80	1.07–3.01	**0.002**	3.98	1.65–9.59
>70 years	75						
Preoperative treatment group							
Short‐course RT	69	0.331	0.73	0.39–1.37	0.783	1.15	0.42–3.13
Long‐course (C)RT	31	0.431	1.31	0.67–2.59	**0.040**	3.19	1.05–9.66
No (C)RT (ref)	38						
CIP2A MICI							
≤Median (ref)	78	0.966	0.99	0.56–1.73	**0.014**	3.02	1.26–7.26
>Median	60						
Disease recurrence							
Yes	39				**<0.0001**	291.8	36.9–2307.1
No (ref)	99						
Postoperative N							
N1	35	0.180	1.49	0.83–2.65	0.831	1.11	0.43–2.83
N2	19	**0.002**	2.88	1.48–5.62	0.945	1.05	0.30–3.60
N0 (ref)	84						
Sex							
Female	62	0.200	0.70	0.40–1.21	0.473	0.75	0.35–1.63
Male (ref)	76						
Lymphovascular invasion							
Yes	43	0.071	1.63	0.96–2.77	0.666	1.19	0.54–2.62
No (ref)	95						
CRM							
≤2 mm	35	0.230	1.40	0.81–2.43	0.297	1.47	0.71–3.02
>2 mm (ref)	103						

CI, confidence interval; CRM, circumferential resection margin; (C)RT, (chemo)radiotherapy; HR, hazard ratio; MICI, most intense cytoplasmic staining index; N, lymph node status; ref, reference.

a Complete data for all the covariates were available for 138 patients.

b Cox regression test.

*P*‐values < 0.05 (in bold) are considered statistically significant.

### The effect of CIP2A knockdown on radiosensitivity

**Figure 2:**
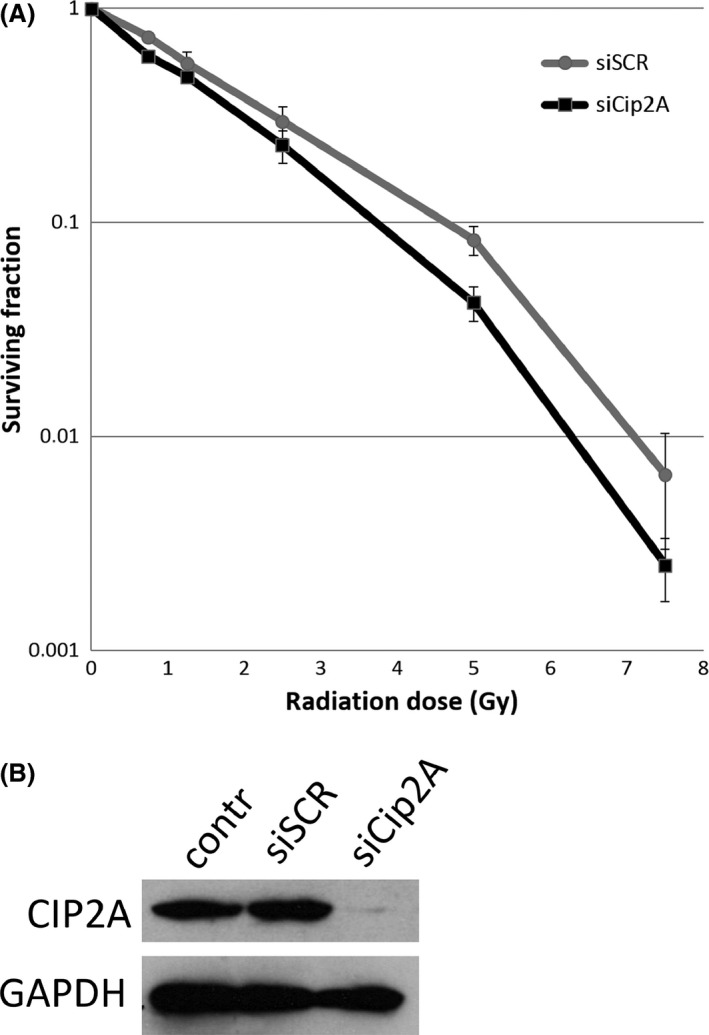
(A) The cell survival curves from the irradiation experiments show the mean surviving fraction ±SE for each radiation dose. The RKO cells treated with CIP2A siRNA (black) were more sensitive to radiation (mean AUC 1.7 ± 0.1 Gy) than the control cells treated with scrambled siRNA (gray) (mean AUC 2.1 ± 0.1 Gy; paired t‐test, *P *=* *0.015). (B) The corresponding Western blot demonstrates the reduction in CIP2A protein expression in siRNA‐treated cells (siCIP2A) in comparison with scrambled siRNA‐treated cells (siSCR) and nontreated control cells (contr). GADPH was used as a loading control.

The mean AUC values for *CIP2A* siRNA‐transfected cells and scrambled control were 1.7 ± 0.1 Gy and 2.1 ± 0.1 Gy, respectively. The cells with *CIP2A* knockdown were significantly more sensitive to radiation than the control cells (paired *t*‐test, *P *=* *0.015). The cell survival curves from the irradiation experiments and corresponding Western blot results are presented in Figure 2.

## Discussion

To our knowledge, the association between CIP2A expression and clinical response to radiotherapy has not been previously studied in rectal cancer. We have investigated CIP2A protein levels in rectal adenocarcinomas from patients receiving either short‐ or long‐course preoperative (chemo)radiotherapy or no treatment before surgery. As the majority of rectal tumors have been shown to overexpress CIP2A 40, we chose to use the most intense cytoplasmic staining index (MICI) together with the average cytoplasmic staining index (ACI) instead of only a dichotomous positive vs. negative IHC scoring system. In the long‐course (C)RT group, patients with low‐CIP2A‐expressing tumors were more likely to respond to preoperative treatment as compared to those with high‐CIP2A‐expressing tumors. This was supported by the observation that transfection of RKO CRC cells with *CIP2A* siRNA sensitized them to irradiation and decreased their survival.

Our results are in agreement with previous findings demonstrating a role for CIP2A in promoting intestinal progenitor cell resistance to irradiation and other DNA‐damaging therapies 29. High CIP2A expression has been found to contribute to radioresistance in HNSCC cells 25, whereas CIP2A immunonegative tumors have been shown to respond favorably to cancer therapies 19, 21. Additionally, the depletion of CIP2A transcription by siRNA has been found to significantly increase radiosensitivity in cervical squamous cell carcinoma and hepatocellular carcinoma cell lines 41.

In this study, low CIP2A expression was found to be more common among the most invasive tumors (pT4) than high CIP2A expression. Still, low CIP2A expression level was associated with better treatment response, defined by tumor regression grade, after long‐course (C)RT. Low CIP2A expression level was also found to be linked with higher 36‐month DSS rate of the patients in categorical analysis. Although CIP2A MICI was not associated with survival in the univariate Kaplan–Meier analysis, low CIP2A expression level was found to remain as an independent prognostic factor for increased DSS together with younger age and nonrecurrent disease, while having received long‐course (C)RT as preoperative therapy was associated with reduced DSS. A prognostic role of CIP2A is also supported by several previous studies, which have found an association between low CIP2A expression and increased survival 27.

In CRC, studies investigating CIP2A as a prognostic biomarker have yielded variable results. In one study, no significant difference was found in five‐year DSS between patients having tumors with either weak or strong CIP2A immunostaining. The survival difference remained statistically nonsignificant even when tumors of colon and rectum were examined separately 40. In contrast, high *CIP2A* mRNA levels have been associated with reduced overall survival (OS) among CRC patients 24. A similar relationship has been observed between strong CIP2A protein expression and reduced OS in *KRAS* wild‐type CRC patients after surgical treatment of liver metastases 42.

In this study, CIP2A expression levels before and after preoperative treatment could not be compared because an adequate number of representative pretreatment biopsies were not available. At the time of diagnosis, biopsy material was not required for molecular pathology and thus often obtained in too small amounts for further analyses. However, it would be of interest to study whether the initial pretreatment CIP2A expression level or the magnitude of change in CIP2A expression during (C)RT also associates with radiation response. CIP2A expression after irradiation has been studied in vivo in mouse testis where its transcription or expression levels did not significantly change over the 144‐h observation period 25. This could support the hypothesis that initially low CIP2A expression might result in more pronounced response to preoperative (C)RT. Additionally, our finding that the knockdown of CIP2A expression by siRNA increases the radiosensitivity of CRC cells in vitro implies that there could be a causal connection between the level of CIP2A expression and radiation response. In conclusion, our results suggest that low CIP2A protein expression level is associated with a favorable response to long‐course (C)RT and with increased disease‐specific survival in rectal cancer. This connection between reduced CIP2A expression and radiosensitivity was further demonstrated in cell irradiation experiments. Thus, the validity of CIP2A as a predictive biomarker for preoperative treatment response in rectal cancer warrants further investigation in the pursuit of providing more personalized treatment modalities for rectal cancer patients.

## Conflict of Interest

The authors declare that they have no competing interests.

## Supporting information


**Table S1.** Association of clinicopathologic variables of rectal cancer patients with CIP2A protein expression defined by the average cytoplasmic staining index (*n *=* *198).

## References

[1] Bray, F. , J. S. Ren , E. Masuyer , and J. Ferlay . 2013 Global estimates of cancer prevalence for 27 sites in the adult population in 2008. Int. J. Cancer 132:1133–1145. 22752881 10.1002/ijc.27711

[2] Ferlay, J. , I. Soerjomataram , M. Ervik , R. Dikshit , S. Eser , C. Mathers , M. Rebelo , et al. 2013 GLOBOCAN 2012 v1.0, Cancer Incidence and Mortality Worldwide: IARC CancerBase No. 11. International Agency for Research on Cancer, Lyon, France Available via http://globocan.iarc.fr, accessed on 2 October 2017.

[3] Glimelius, B. , E. Tiret , A. Cervantes , and D. Arnold . 2013 Rectal cancer: ESMO Clinical Practice Guidelines for diagnosis, treatment and follow‐up. Ann. Oncol. 24(Suppl 6):vi81–vi88. 24078665 10.1093/annonc/mdt240

[4] Folkesson, J. , H. Birgisson , L. Påhlman , B. Cedermark , B. Glimelius , and U. Gunnarsson . 2005 Swedish Rectal Cancer Trial: long lasting benefits from radiotherapy on survival and local recurrence rate. J. Clin. Oncol. 23:5644–5650. 16110023 10.1200/JCO.2005.08.144

[5] Peeters, K. C. , C. A. Marijnen , I. D. Nagtegaal , E. K. Kranenbarg , H. Putter , T. Wiggers , et al. 2007 The TME trial after a median follow‐up of 6 years: increased local control but no survival benefit in irradiated patients with resectable rectal carcinoma. Ann. Surg. 246:693–701. 17968156 10.1097/01.sla.0000257358.56863.ce

[6] Shin, J. S. , T. G. Tut , V. Ho , and C. S. Lee . 2014 Predictive markers of radiotherapy‐induced rectal cancer regression. J. Clin. Pathol. 67:859–864. 25118294 10.1136/jclinpath-2014-202494

[7] Ghadimi, B. M. , M. Grade , M. J. Difilippantonio , S. Varma , R. Simon , C. Montagna , et al. 2005 Effectiveness of gene expression profiling for response prediction of rectal adenocarcinomas to preoperative chemoradiotherapy. J. Clin. Oncol. 23:1826–1838. 15774776 10.1200/JCO.2005.00.406 PMC4721601

[8] Bao, S. , Q. Wu , R. E. McLendon , Y. Hao , Q. Shi , A. B. Hjelmeland , et al. 2006 Glioma stem cells promote radioresistance by preferential activation of the DNA damage response. Nature 444:756–760. 17051156 10.1038/nature05236

[9] Watanabe, T. , Y. Komuro , T. Kiyomatsu , T. Kanazawa , Y. Kazama , J. Tanaka , et al. 2006 Prediction of sensitivity of rectal cancer cells in response to preoperative radiotherapy by DNA microarray analysis of gene expression profiles. Can. Res. 66:3370–3374. 10.1158/0008-5472.CAN-05-3834 16585155

[10] Negri, F. V. , N. Campanini , R. Camisa , F. Pucci , S. Bui , G. Ceccon , et al. 2008 Biological predictive factors in rectal cancer treated with preoperative radiotherapy or radiochemotherapy. Br. J. Cancer 98:143–147. 18087284 10.1038/sj.bjc.6604131 PMC2359706

[11] Kuremsky, J. G. , J. E. Tepper , and H. L. McLeod . 2009 Biomarkers for response to neoadjuvant chemoradiation for rectal cancer. Int. J. Radiat. Oncol. Biol. Phys. 74:673–688. 19480968 10.1016/j.ijrobp.2009.03.003

[12] Agostini, M. , K. P. Janssen , I. J. Kim , E. D'Angelo , S. Pizzini , A. Zangrando , et al. 2015 An integrative approach for the identification of prognostic and predictive biomarkers in rectal cancer. Oncotarget 6:32561–32574. 26359356 10.18632/oncotarget.4935 PMC4741712

[13] Carpinetti, P. , E. Donnard , F. Bettoni , P. Asprino , F. Koyama , A. Rozanski , et al. 2015 The use of personalized biomarkers and liquid biopsies to monitor treatment response and disease recurrence in locally advanced rectal cancer after neoadjuvant chemoradiation. Oncotarget 6:38360–38371. 26451609 10.18632/oncotarget.5256 PMC4742005

[14] Chang, L. , P. Graham , J. Hao , J. Ni , J. Deng , J. Bucci , et al. 2015 Cancer stem cells and signaling pathways in radioresistance. Oncotarget 7:11002–11017. 10.18632/oncotarget.6760 PMC4905454 26716904

[15] Lim, S. H. , W. Chua , C. Henderson , W. Ng , J. S. Shin , L. Chantrill , et al. 2015 Predictive and prognostic biomarkers for neoadjuvant chemoradiotherapy in locally advanced rectal cancer. Crit. Rev. Oncol. Hematol. 96:67–80. 26032919 10.1016/j.critrevonc.2015.05.003

[16] Molinari, C. , F. Matteucci , P. Caroli , and A. Passardi . 2015 Biomarkers and molecular imaging as predictors of response to neoadjuvant chemoradiotherapy in patients with locally advanced rectal cancer. Clin. Colorectal Cancer 14:227–238. 26170142 10.1016/j.clcc.2015.05.014

[17] Junttila, M. R. , P. Puustinen , M. Niemelä , R. Ahola , H. Arnold , T. Böttzauw , et al. 2007 CIP2A inhibits PP2A in human malignancies. Cell 130:51–62. 17632056 10.1016/j.cell.2007.04.044

[18] Khanna, A. , J. E. Pimanda , and J. Westermarck . 2013 Cancerous inhibitor of protein phosphatase 2A, an emerging human oncoprotein and a potential cancer therapy target. Can. Res. 73:6548–6553. 10.1158/0008-5472.CAN-13-1994 24204027

[19] Laine, A. , H. Sihto , C. Côme , M. T. Rosenfeldt , A. Zwolinska , M. Niemelä , et al. 2013 Senescence sensitivity of breast cancer cells is defined by positive feedback loop between CIP2A and E2F1. Cancer Discov. 3:182–197. 23306062 10.1158/2159-8290.CD-12-0292 PMC3572190

[20] Khanna, A. , C. Böckelman , A. Hemmes , M. R. Junttila , J. P. Wiksten , M. Lundin , et al. 2009 MYC‐dependent regulation and prognostic role of CIP2A in gastric cancer. J. Natl Cancer Inst. 101:793–805. 19470954 10.1093/jnci/djp103

[21] Böckelman, C. , H. Lassus , A. Hemmes , A. Leminen , J. Westermarck , C. Haglund , et al. 2011 Prognostic role of CIP2A expression in serous ovarian cancer. Br. J. Cancer 105:989–995. 21897396 10.1038/bjc.2011.346 PMC3185957

[22] Dong, Q. Z. , Y. Wang , X. J. Dong , Z. X. Li , Z. P. Tang , Q. Z. Cui , et al. 2011 CIP2A is overexpressed in non‐small cell lung cancer and correlates with poor prognosis. Ann. Surg. Oncol. 18:857–865. 20842459 10.1245/s10434-010-1313-8

[23] Lucas, C. M. , R. J. Harris , A. Giannoudis , M. Copland , J. R. Slupsky , and R. E. Clark . 2011 Cancerous inhibitor of PP2A (CIP2A) at diagnosis of chronic myeloid leukemia is a critical determinant of disease progression. Blood 117:6660–6668. 21490338 10.1182/blood-2010-08-304477

[24] Wiegering, A. , C. Pfann , F. W. Uthe , C. Otto , L. Rycak , U. Mäder , et al. 2013 CIP2A influences survival in colon cancer and is critical for maintaining Myc expression. PLoS ONE 8:e75292. 24098375 10.1371/journal.pone.0075292 PMC3788051

[25] Ventelä, S. , E. Sittig , L. Mannermaa , J. A. Mäkelä , J. Kulmala , E. Löyttyniemi , et al. 2015 CIP2A is an Oct4 target gene involved in head and neck squamous cell cancer oncogenicity and radioresistance. Oncotarget 6:144–158. 25474139 10.18632/oncotarget.2670 PMC4381584

[26] Côme, C. , A. Laine , M. Chanrion , H. Edgren , E. Mattila , X. Liu , et al. 2009 CIP2A is associated with human breast cancer aggressivity. Clin. Cancer Res. 15:5092–5100. 19671842 10.1158/1078-0432.CCR-08-3283

[27] Khanna, A. , and J. E. Pimanda . 2016 Clinical significance of cancerous inhibitor of protein phosphatase 2A in human cancers. Int. J. Cancer 138:525–532. 25628223 10.1002/ijc.29431

[28] Ventelä, S. , C. Côme , J. A. Mäkelä , R. M. Hobbs , L. Mannermaa , M. Kallajoki , et al. 2012 CIP2A promotes proliferation of spermatogonial progenitor cells and spermatogenesis in mice. PLoS ONE 7:e33209. 22461891 10.1371/journal.pone.0033209 PMC3312892

[29] Myant, K. , X. Qiao , T. Halonen , C. Côme , A. Laine , M. Janghorban , et al. 2015 Serine 62‐phosphorylated MYC associates with nuclear lamins and its regulation by CIP2A is essential for regenerative proliferation. Cell Rep. 12:1019–1031. 26235622 10.1016/j.celrep.2015.07.003 PMC4535171

[30] Glimelius, B. , L. Påhlman , and A. Cervantes . 2010 Rectal cancer: ESMO Clinical Practice Guidelines for diagnosis, treatment and follow‐up. Ann. Oncol. 21(Suppl 5):v82–v86. 20555109 10.1093/annonc/mdq170

[31] Sobin, L. H. , and C. Wittekind . 2002 Colon and rectum Pp. 72–76 *in* Sobin L. H., Wittekind C., eds. TNM classification of malignant tumours, 6th ed UICC. Wiley, New York, NY.

[32] Dworak, O. , L. Keilholz , and A. Hoffmann . 1997 Pathological features of rectal cancer after preoperative radiochemotherapy. Int. J. Colorectal Dis. 12:19–23. 9112145 10.1007/s003840050072

[33] Rödel, C. , P. Martus , T. Papadoupolos , L. Füzesi , M. Klimpfinger , R. Fietkau , et al. 2005 Prognostic significance of tumor regression after preoperative chemoradiotherapy for rectal cancer. J. Clin. Oncol. 23:8688–8696. 16246976 10.1200/JCO.2005.02.1329

[34] Korkeila, E. , K. Talvinen , P. M. Jaakkola , H. Minn , K. Syrjänen , J. Sundström , et al. 2009 Expression of carbonic anhydrase IX suggests poor outcome in rectal cancer. Br. J. Cancer 100:874–880. 19240720 10.1038/sj.bjc.6604949 PMC2661792

[35] Avoranta, S. T. , E. A. Korkeila , K. J. Syrjänen , S. O. Pyrhönen , and J. T. Sundström . 2012 Lack of CD44 variant 6 expression in rectal cancer invasive front associates with early recurrence. World J. Gastroenterol. 18:4549–4556. 22969228 10.3748/wjg.v18.i33.4549 PMC3435780

[36] Soo Hoo, L. , J. Y. Zhang , and E. K. Chan . 2002 Cloning and characterization of a novel 90 kDa ‘companion’ auto‐antigen of p62 overexpressed in cancer. Oncogene 21:5006–5015. 12118381 10.1038/sj.onc.1205625

[37] Lipponen, P. , and Y. Collan . 1992 Simple quantitation of immunohistochemical staining positivity in microscopy for histopathology routine. Acta Stereol. 11:125–132.

[38] Ålgars, A. , T. Avoranta , P. Österlund , M. Lintunen , J. Sundström , T. Jokilehto , et al. 2014 Heterogeneous EGFR gene copy number increase is common in colorectal cancer and defines response to anti‐EGFR therapy. PLoS ONE 9:e99590. 24940619 10.1371/journal.pone.0099590 PMC4062406

[39] Pekkola‐Heino, K. , J. Kulmala , S. Grénman , T. E. Carey , and R. Grénman . 1989 Radiation response of vulvar squamous cell carcinoma (UM‐SCV‐1A, UM‐SCV‐1B, UM‐SCV‐2, and A‐431) cells in vitro. Can. Res. 49:4876–4878. 2758419

[40] Böckelman, C. , S. Koskensalo , J. Hagström , M. Lundin , A. Ristimäki , and C. Haglund . 2012 CIP2A overexpression is associated with c‐Myc expression in colorectal cancer. Cancer Biol. Ther. 13:289–295. 22310977 10.4161/cbt.18922

[41] Huang, C. , C. Wei , K. Chen , H. Chen , A. Cheng , and K. Chen . 2012 Bortezomib enhances radiation‐induced apoptosis in solid tumors by inhibiting CIP2A. Cancer Lett. 317:9–15. 22085493 10.1016/j.canlet.2011.11.005

[42] Chen, K. F. , C. C. Yen , J. K. Lin , W. S. Chen , S. H. Yang , J. K. Jiang , et al. 2015 Cancerous inhibitor of protein phosphatase 2A (CIP2A) is an independent prognostic marker in wild‐type KRAS metastatic colorectal cancer after colorectal liver metastasectomy. BMC Cancer 15:301. 25896895 10.1186/s12885-015-1300-3 PMC4404594

