# Supplementary material for: Protein phosphatase 2A (PP2A) inhibitor CIP2A indicates resistance to radiotherapy in rectal cancer
Source: Cancer Med. 2018 Feb 14;7(3):698–706. doi: 10.1002/cam4.1361 (PMC5852361; doi:10.1002/cam4.1361)
Supplement: Supplementary file 1 — Table S1. Association of clinicopathologic variables of rectal cancer patients with CIP2A protein expression defined by the average cytoplasmic staining index (n = 198). [file CAM4-7-698-s001.docx]

| **Supplementary table 1.** Association of clinicopathologic variables of rectal cancer patients with CIP2A protein expression defined by the average cytoplasmic staining index (*n* = 198). | | | | | | |
| --- | --- | --- | --- | --- | --- | --- |
|  | | **CIP2A average cytoplasmic staining index** | | | | |
|  | | Below median, *n* (%) | Above median, *n* (%) | *P*-value¶ |  |  |
| Grade† | |  |  |  |  |  |
| GI | | 21 (21.2) | 9 (9.5) | **0.050** |  |  |
| G2 | | 59 (59.6) | 70 (73.7) |  |  |  |
| G3 | | 19 (19.2) | 16 (16.8) |  |  |  |
| Post-treatment tumour regression‡ | |  |  |  |  |  |
| Poor | | 16 (50.0) | 10 (100.0) | **0.007** |  |  |
| Moderate/excellent | | 16 (50.0) | 0 (0) |  |  |  |
| DSS§ | |  |  |  |  |  |
| ≥ 36 months | | 81 (90.0) | 69 (79.3) | 0.060 |  |  |
| < 36 months | | 9 (10.0) | 18 (20.7) |  |  |  |
|  | |  |  |  |  |  |
| †Excluding four tumours that could not be graded. | | | | | | |
| ‡Assessed only after long-course (chemo)radiotherapy | | | | | | |
| §Disease-specific survival, alive vs. death of disease | | |  |  |  |  |
| ¶Fisher’s exact test. | | | | | | |
|  |  | |  |  |  |  |
